# Is the Zero-P Spacer Suitable for 3-Level Anterior Cervical Discectomy and Fusion Surgery in Terms of Sagittal Alignment Reconstruction: A Comparison Study with Traditional Plate and Cage System

**DOI:** 10.3390/brainsci12111583

**Published:** 2022-11-19

**Authors:** Jing Guo, Weiming Jin, Yan Shi, Zhiping Guan, Jian Wen, Yongcan Huang, Binsheng Yu

**Affiliations:** Department of Spine Surgery, Peking University Shenzhen Hospital, Shenzhen 518036, China

**Keywords:** cervical spine, anterior cervical discectomy and fusion, Zero-P spacer, sagittal alignment, cervical curvature

## Abstract

The Zero-P spacer was primarily developed aiming to reduce the morbidity associated with the traditional anterior cervical plate. During the past decade, many authors have reported the use of Zero-P spacers for anterior cervical discectomy and fusion (ACDF) of one or two segments. Nevertheless, there is still a paucity of knowledge on the safety and feasibility of using Zero-P spacers for 3-level fixation. The objective of this study was to investigate the clinical and radiological outcomes, with a focus on the sagittal alignment reconstruction of 3-level ACDF surgery using Zero-P spacers versus those using a traditional plate and cage system. From Sep 2013 to Aug 2016, a total of 44 patients who received 3-level ACDF surgery due to cervical spondylotic myelopathy were recruited. The Zero-P spacer was used in 23 patients (group ZP) and the traditional plate and cage system in 21 (group PC). Clinical outcomes were analyzed by Neck Disability Index (NDI) and Japanese Orthopedic Association (JOA) scores, and dysphagia was evaluated using the Bazaz score. Radiological outcomes, including fusion rate, adjacent segment degeneration (ASD), and especially changes in cervical sagittal alignment, were analyzed. The NDI and JOA scores did not differ significantly between the two groups postoperatively (*p* > 0.05); however, there was significantly less dysphagia in patients using Zero-P spacers at the 3- and 6-month follow-up (*p* < 0.05). At the 24-month follow-up, the fusion rate and ASD were similar between the two groups (*p* > 0.05). Interestingly, patients using Zero-P spacers had a significantly lower postoperative C2-7 Cobb angle and fused segment Cobb angle, compared to those using a traditional plate and cage system (*p* < 0.05); meanwhile, the fused segment disc wedge was also found to be significantly smaller in patients using Zero-P spacers after surgery (*p* < 0.05). Moreover, we further divided patients into subgroups according to their cervical lordosis. In patients with a preoperative C2-7 Cobb angle ≤ 10°, significantly less cervical and local lordosis, as well as disc wedge, were seen in group ZP after surgery (*p* < 0.05), while in others with a preoperative C2-7 Cobb angle > 10°, no significant difference in postoperative changes of the cervical sagittal alignment was seen between group ZP and group PC (*p* > 0.05). Zero-P spacers used in 3-level ACDF surgery could provide equivalent clinical outcomes and a lower rate of postoperative dysphagia, compared to the traditional plate and cage system. However, our results showed that it was inferior to the cervical plate in terms of sagittal alignment reconstruction for 3-level fixation. We recommend applying Zero-P spacers for 3-level ACDF in patients with good preoperative cervical lordosis (C2-7 Cobb angle > 10°), in order to restore and maintain physiological curvature of the cervical spine postoperatively.

## 1. Introduction

Cervical spondylotic myelopathy (CSM) is a common degenerative disease of the cervical spine, causing neurologic deficits with or without arm pain [1,2]. Anterior cervical discectomy and fusion (ACDF), first reported by Smith [3] and Cloward [4] in the 1950s, has nowadays been widely applied and is typically considered as a standard procedure in treating patients with CSM [5,6]. Conventionally, anterior cervical plates are used in most ACDF surgeries, in terms of providing supplemental fixation and the avoiding migration of the intervertebral cages [7,8]. Many literatures have demonstrated that the anterior plate and cage system can achieve a higher fusion rate and a lower rate of implant failure [9,10]. However, plate-associated problems, such as dysphagia after surgery, adjacent segment degeneration, and plate shifting, have been reported and raised the surgeons’ concern, especially in multilevel cases [7,8,9,10].

The Zero-P spacer is a novel zero-profile, stand-alone, and self-locking cervical interbody cage, which was primarily designed to reduce the complications associated with the traditional anterior cervical plate [11,12,13,14,15]. During the past few years, many scholars have reported the use of Zero-P spacers in treating 1- or 2-level CSM [16,17,18,19,20,21]; thus, satisfactory clinical and radiological outcomes were documented [22,23,24,25,26,27]. However, there is still a paucity of knowledge on the safety and feasibility of using a Zero-P spacer for 3-level fixation, with respect to the sagittal cervical alignment reconstruction. In 2017, Chen et al. [28] compared the mid-term results of 3-level ACDF between Zero-P spacer and the traditional cervical plate, and reported that, although the Zero-P spacer was similar to the traditional cervical plate in clinical outcomes, it was inferior in the restoration of cervical lordosis and could not reconstruct a better sagittal cervical alignment in 3-level fixation. Thereafter, Sun et al. [29] and Xiao et al. [30] also reported that, for 3-level ACDF surgery, a Zero-P spacer was not as comparable as the plate-cage system in maintaining postoperative cervical alignment. As a matter of fact, in our clinical practice, we have noticed that in some patients with a certain preoperative cervical lordosis, good sagittal cervical alignment reconstruction can be achieved for 3-level ACDF even by using Zero-P spacers. Therefore, we hypothesized that the preoperative cervical curvature might be of some clinical relevance in predicting the postoperative sagittal cervical alignment for 3-level ACDF surgery.

The objective of this study was to investigate clinical and radiological outcomes, with a specific focus on sagittal cervical alignment reconstruction, of 3-level ACDF surgery using Zero-P spacers versus those using the traditional plate and cage system. We also attempted to identify whether preoperative cervical lordosis could be used as an indicator for sagittal cervical alignment reconstruction in the same procedure.

## 2. Materials and Methods

This study was approved by the institutional ethics committee of our hospital. From September 2013 to August 2016, a total of 44 patients (24 males and 20 females) with degenerative disc disease were recruited into this study. All patients received 3-level ACDF surgery from C3 to C7 for CSM after conservative treatment for at least 3 months. The selection of the implant for surgery (Zero-P or anterior plate system) was based on the patients’ willingness. The Zero-P spacer was used in 23 patients (group ZP), and the traditional plate and cage system was used in 21 patients (group PC).

**Figure 1:**
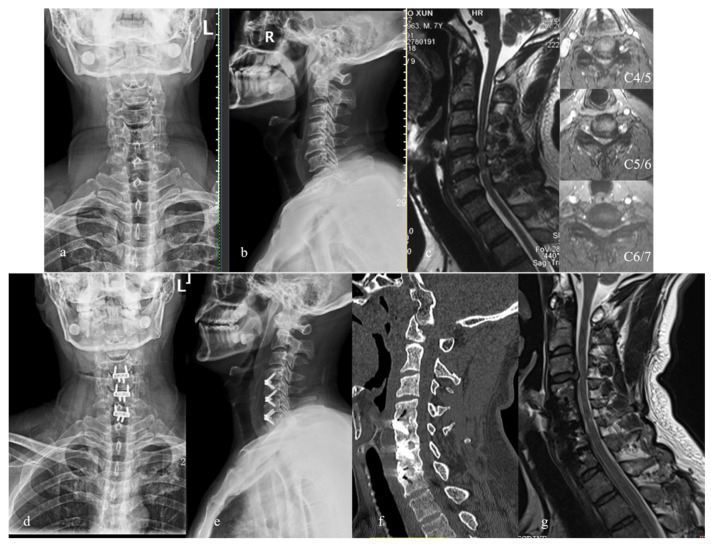
A 54-year-old male with walking disturbance and clumsy hands for 5 years and radiating pain in the left arm for 6 months. (**a**,**b**) Preoperative x-ray films; (**c**) preoperative MRI showing C4-C7 disc herniation compressing the spinal cord, and stenosis on the left nerve root canals; (**d**,**e**) postoperative X-ray films showing C4-C7 ACDF with Zero-P spacers; (**f**) CT scan at 2-year follow-up showing good implant position and bone fusion; (**g**) MRI at 2-year follow-up showing ASD at C3/4; however, there was no complaint of any symptoms and observation was implemented.

**Figure 2:**
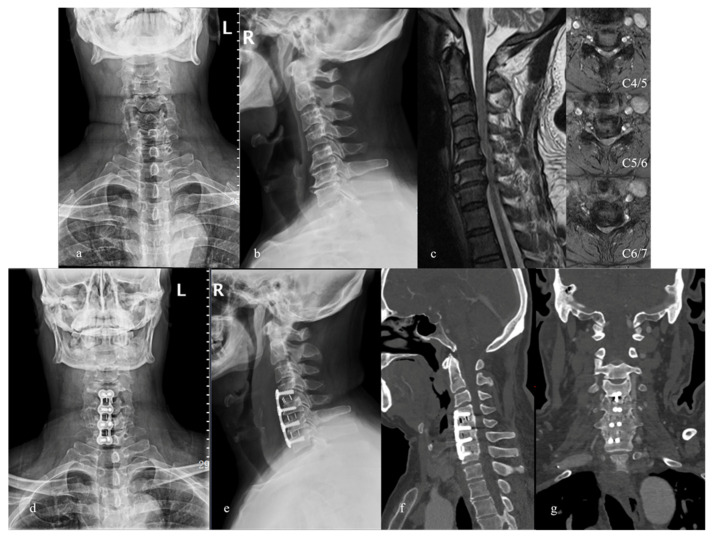
A 53-year-old male with upper limb numbness and clumsiness as well as walking disturbance for 1 year. (**a**,**b**) Preoperative X-ray films; (**c**) preoperative MRI showing spinal canal stenosis with C4-C7 disc herniation compressing the spinal cord; (**d**,**e**) postoperative X-ray films showing C4-C7 ACDF with plate-cage system; (**f**,**g**) CT scans at 3-year follow-up showing good implant position and bone fusion.

All surgeries were performed by the same surgeon (BS.Y.). The standard anterior Smith-Robinson approach was performed. First, the thorough decompression and removal of the degenerated discs were accomplished; after trialing, suitable Zero-P spacers (DePuy Synthes Spine, USA) were selected and then inserted into the intervertebral space in group ZP (Figure 1), while in group PC, appropriate interbody cages were inserted and a pre-bent plate (D&J Medical, Changzhou, China) was applied with locking screws for the fixation (Figure 2). Finally, anteroposterior and lateral fluoroscopies were preformed to confirm the positioning of the implants.

All patients had a minimal postoperative follow-up of 24 months. At each follow-up, neutral/flexion/extension X-rays were obtained for radiological assessment. Radiographic fusion was determined by the interspinous process method [31], and solid fusion was regarded as a motion difference of less than 1-mm on the lateral flexion-extension X-ray radiographs. Adjacent segment disease (ASD) was defined by the following radiological evidences: (1) formation or increased anterior osteophytes; (2) new or increased disc space narrowing (>30%); (3) new or increased anterior longitudinal ligament calcification; (4) growth of radial osteophytes [32].

**Figure 3:**
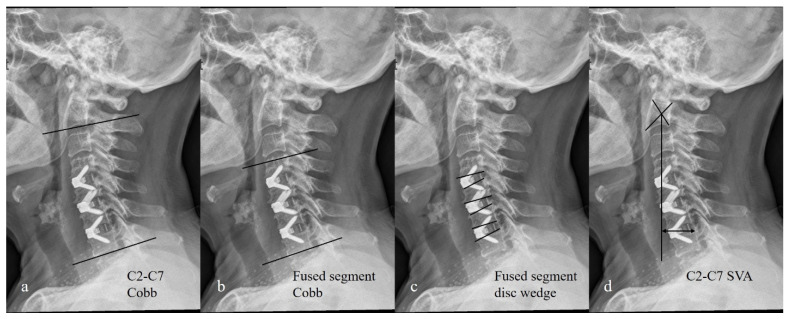
Illustration showing the method of cervical sagittal alignment measurements. (**a**) C2-C7 Cobb angle; (**b**) fused segment Cobb angle (in this case, C4-C7); (**c**) fused segment disc wedge (in this case, C4/5+C5/6+C6/7); (**d**) C2-C7 sagittal vertical axis.

The sagittal alignment of the cervical spine was measured on lateral X-ray films. Cervical lordosis was measured as the angle between the lower endplate of C2 and the lower endplate of C7 (C2-C7 Cobb) (Figure 3a). If the lower endplate of the C7 vertebra was blurry or invisible, the upper endplate of C7 or the lower endplate of C6 was chosen. The fused segment Cobb angle was measured by drawing two lines between the upper and lower endplates of the cranial and caudal vertebrae in the fused segment (Figure 3b). The fused segment disc wedge was calculated by adding up the Cobb angles of the three operated discs (Figure 3c). The C2-C7 sagittal vertical axis (SVA) was measured as the direct distance from the plumb line through the centroid of the C2 vertebral body to the posterior-superior corner of C7 (Figure 3d). These assessments were performed twice for each patient by two surgeons independently (J.G. and W.J.). All measurements were performed on the picture archiving and communication system (PACS) of our hospital (EW ViewerPro, Version 4.9.0.5001).

Clinical outcomes were assessed by the Japanese Orthopedic Association (JOA) score and Neck Disability Index (NDI) questionnaires. The incidence of dysphagia was evaluated by using the Bazaz system at 48 h postoperatively and at the 3-, and 6-month follow-ups (Table 1). Functional and radiological assessments were performed preoperatively, 3 months, and 6 months after surgery, and then yearly.

Statistical analysis was performed using SPSS for Windows, Version 16.0 (SPSS Inc.). The independent-samples t tests were performed to compare measurement data between the two groups, and chi-square tests were applied to analyze enumeration data. Statistical significance was regarded as a *p* value < 0.05.

## 3. Results

Operative data were presented in Table 2. There was no significant difference in operation time and intraoperative blood loss between the two groups. Table 3 showed the results of clinical outcomes before and after surgery. All patients experienced the improvement of symptoms after the surgery. Both groups demonstrated a significant improvement in NDI and JOA scores after surgery, but there was no significant difference between group ZP and group PC. One patient in group PC developed right C5 nerve root palsy immediately after surgery, and conservative treatment was performed. The patient recovered completely by the 3-month follow-up. There was no neurological deficit, implant failure, wound infection, or cerebrospinal fluid (CSF) leakage in both groups.

Forty-eight hours after surgery, a total of eight patients in group ZP developed mild dysphagia; while in group PC, dysphagia was also observed in eight patients (four mild, four moderate). At the 3-month follow-up, only one patient in group ZP still complained of mild dysphagia, and this symptom disappeared by the 6-month follow-up. In group PC, moderate dysphagia was observed in two patients and mild dysphagia in four at the 3-month follow-up. Of these six patients, four still had mild dysphagia at the 6-month follow-up. The incidences of dysphagia were found to be significantly higher in group PC than those in group ZP at the 3- and 6-month follow-ups.

Radiological outcomes are demonstrated in Table 4. The rate of fusion at the 24-month follow-up was 91.3% (21/23) in group ZP and 95.2% (20/21) in group PC (*p* = 0.605), whereas, ASD was found in three group ZP patients and three group PC patients at the 24-month follow-up (*p* = 0.905). However, none of them required revision surgery.

The mean C2-C7 Cobb angle, fused segment Cobb angle, and fused segment disc wedge were comparable between the two groups preoperatively (Table 4). At the last follow-up, the average C2-C7 Cobb angle was 12.5 ± 5.7 degrees in group ZP and 18.6 ± 6.9 degrees in group PC (*p* = 0.023); the average Cobb angles of the fused segments were 9.5 ± 6.6 degrees in group ZP and 13.9 ± 7.4 in group PC (*p* = 0.025); the mean fused segment disc wedge was 8.1 ± 6.8 degrees in group ZP and 13.6 ± 7.5 in group PC (*p* = 0.021). There was obviously more improvement in cervical sagittal alignment (C2-C7 Cobb angle, fused segment Cobb angle, fused segment disc wedge) in group PC than group ZP after the operation. However, no significant difference was found in C2-C7 SVA between the two groups pre- and postoperatively.

Finally, we further divided patients into subgroups according to the preoperative cervical lordosis (C2-C7 Cobb angle) (Table 5). Interestingly, we found that, in patients with a preoperative C2-C7 Cobb angle ≤ 10°, significantly less cervical and fused segment lordosis, as well as fused segment disc wedge, were seen in group ZP postoperatively, as compared to group PC (*p* < 0.05); while in others with a preoperative C2-C7 Cobb angle > 10°, no significant difference in postoperative changes of cervical sagittal alignment was seen between group ZP and group PC (*p* > 0.05).

## 4. Discussion

Over the past 30 years, ACDF has generally become a “main-stream” surgical technique for treating multilevel CSM, which possesses the advantages of easy surgical exposure, little intraoperative bleeding, and quick postoperative recovery [5,6]. Conventionally, the application of an anterior cervical plate and cage system in ACDF has led to the acknowledgment of several pros and cons [5,33]. The former included greater initial stability, a better recovery of cervical lordosis, and a higher fusion rate [5,33]. Meanwhile, plate-associated complications, such as dysphagia, implant failure, and ASD, have aroused great concern [5,33]. In the treatment of multilevel CSM, extensive exposure is mandatory for plate and screw installation, which not only increases iatrogenic trauma, but also makes the surgical procedure technically demanding [25]. A Zero-P spacer is a stand-alone anchored device which is primarily designed to reduce complications related to the anterior cervical plate system, while maintaining the benefits of interbody fusion and fixation [11,12]. It requires a smaller dissection and avoids implant contact with the anterior soft tissue, making it less prone to complications compared with the traditional plate construct [13,14,15]. In spite of the increased use of Zero-P in CSM nowadays, there is still a debate on its application for multilevel ACDF, especially for those with three levels or more. The current study provided clinical and radiological estimates to determine which device (the Zero-P spacer versus the traditional plate system) is more suitable for 3-level ACDF surgery, with a specific focus on cervical sagittal alignment and balance.

It has been widely accepted that cervical lordosis plays an important role in maintaining sagittal head and spinal balance [34]. Compromise on this lordotic curvature of the cervical spine, such as hypolordosis or kyphosis, is usually associated with neck pain, disability, and cervical disc degeneration [35]. Song et al. [8] compared the efficacy of ACDF with a cage alone or a cage and plate construct in a total of 78 patients. They found that the use of a plate in 1- or 2-level ACDF resulted in a better lordotic alignment, a higher fusion rate, a lower subsidence rate, and a lower complication rate than that of cage alone. In 2017, Chen compared the mid-term results of 3-level ACDF between the Zero-P spacer and the traditional plate and cage system [28]. In their study, the C2-C7 Cobb angle and the fused segment Cobb angle were significantly greater in the plate group than those in the Zero-P group after surgery. They demonstrated that the use of a pre-bent plate could result in a good reconstruction of the sagittal alignment, despite a poor preoperative curve. In 2020, Sun et al. [29] reviewed 61 cases (Zero-P 27 vs. plate-cage 34) undergoing 3-level ACDF with a follow-up of 5-years. They found that, during the postoperative follow-up, the Zero-P spacer demonstrated a higher loss of correction on the disc height and C2-C7 Cobb angle compared to the plate and cage system. Recently, Xiao et al. [30] studied the impact of 3-level ACDF on the occipito-atlantoaxial complex between the Zero-P spacer and the plate-cage system. In the Zero-P group, the restoration of cervical lordosis (C2-C7 Cobb angle) was significantly lost at the 1-year follow-up compared with the plate-cage group. These previous studies have implied that special caution should be taken with respect to the cervical sagittal alignment while using a Zero-P spacer for multilevel ACDF surgery.

In the present study, when speaking generally, the changes of the cervical sagittal alignment using a Zero-P spacer or a plate and cage system were similar to those in previously published papers [8,28,29,30]. For both groups, the C2-C7 Cobb angle, the fused segment Cobb angle, as well as the fused segment disc wedge were found to be significantly improved immediately after surgery, and then these changes gradually decreased during follow-ups (Table 4). This phenomenon could be attributed to the subtle diminishing of disc height and wedging among the fused segments [29,30]. Although we did not observe any case with obvious cage subsidence in our series, this trivial change at each disc level may add up to a certain extent, causing the loss of correction in the cervical sagittal alignment.

**Figure 4:**
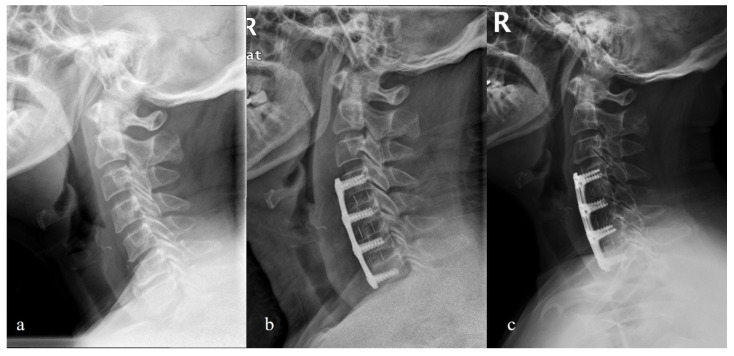
Lateral radiographs of one patient receiving anterior plate and cage system. (**a**) Preoperative C2-C7 Cobb angle was 10 degrees; (**b**) postoperative C2-C7 Cobb angle increased to 22 degrees; (**c**) the C2-C7 Cobb angle maintained 18 degrees at the 3-year follow-up.

**Figure 5:**
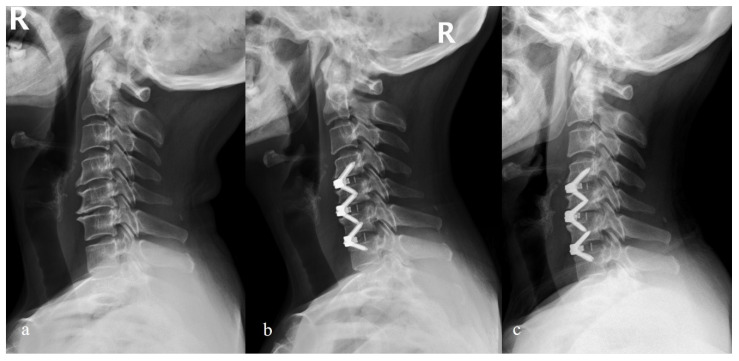
Lateral radiographs of one patient receiving Zero-P spacer system. (**a**) Preoperative C2-C7 Cobb angle was 5 degrees; (**b**) postoperative C2-C7 Cobb angle decreased to -5 degrees (kyphotic); (**c**) the C2-C7 Cobb angle was 0 degrees at the 2-year follow-up.

**Figure 6:**
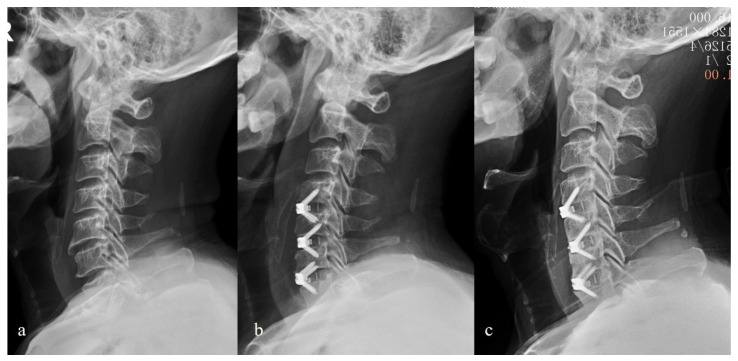
Lateral radiographs of one patient receiving Zero-P spacer system. (**a**) Preoperative C2-C7 Cobb angle was 15 degrees; (**b**) postoperative C2-C7 Cobb angle improved to 22 degrees; (**c**) the C2-C7 Cobb angle was 19 degrees at the 2-year follow-up.

At last follow-up, not only the C2-C7 Cobb angle and the fused segment Cobb angle, but also the fused segment disc wedge were found to be significantly greater in the plate group (Table 4). These findings implicated that the anterior cervical plate might have a better ability than the Zero-P spacer to reform the vertebral alignment in the sagittal plane, probably due to its pre-bent-shape nature; meanwhile, the locking screws could also help to reinforce the realignment process by maintaining disc wedging over the fused segments (Figure 4). However, when we divided patients into subgroups according to their preoperative cervical lordosis, this abovementioned advantage of the plate system diminished. For patients with a preoperative C2-C7 Cobb angle of more than 10 degrees, Zero-P spacers were found to have a similar capability to the plate system in restoring good cervical curvature (Table 5) (Figure 5 and Figure 6). This could provide some extent of clinical relevance for surgeons to consider when planning on using Zero-P spacers for 3-level ACDF surgery.

Of all of the complications related to the anterior cervical plate system, dysphagia and tracheoesophageal lesions are most frequent and specific, reportedly with an incidence of up to 30% during the first 3 months post-operation [36]. The possible cause of postoperative dysphagia is generally regarded as mechanical irritation to the esophagus by direct contact with a plate of a certain thickness [16]. In addition, the anterior soft tissue swelling may also contribute to dysphagia postoperatively [28]. The Zero-P spacer utilizes an integrated, mini-sized plate containing four screws for endplate fixation, which enables it to be inserted completely within the disc space, avoiding the mechanical irritation of the esophagus [12]. Due to its compact design, the need for extensive soft tissue dissection and osteophyte resection is decreased, leading to less damage to the anterior tissue, thus preventing dysphagia [13]. In our results, the rates of dysphagia were comparable between group ZP (34.8%) and group PC (38.1%) 48 h after surgery (*p* > 0.05). However, a significantly lower rate of dysphagia was observed in group ZP (4.3%) than in group PC (28.6%) at the 3-month follow-up (*p* < 0.05). At the 6-month follow-up, symptoms of dysphagia had resolved completely in group ZP; while there were still four patients (19.0%) complaining in group PC (*p* < 0.05).

In the historical view, ACDF without plating is not a “new story”. Before the advent of the anterior cervical plate, stand-alone interbody cages without self-locking features and autogenous bone grafts were primarily used in ACDF surgery. Although this technique has been rarely performed worldwide nowadays, it still prevails in some regions. Pinder et al. [37] reported 46 cases using the Solis cage alone and 15 cases combined with anterior plate fixation. The extent and rate of cage subsidence were both greater in the cage alone group, compared to the plate group. Lee et al. [38] found that, although the overall clinical outcomes were comparable between ACDF with and without plating, plating did play a role in preventing segmental kyphosis and cage subsidence, thus promoted bone fusion. Kwon et al. [39] and Fujibayashi et al. [40] both claimed that ACDF with a plate could restore more cervical lordosis and was more effective in preserving the acquired alignment compared with ACDF using cages alone. Oliver et al. [6] conducted a systematic review and meta-analysis for the comparison of postoperative clinical and radiological outcomes, following ACDF with and without plate fixation. They concluded that ACDF without plating had a higher rate of cage subsidence, a lower fusion rate, and less improvement in neck pain; however, no significant differences in postoperative dysphagia or NDI scores were found. Although potential pitfalls have been documented regarding stand-alone interbody cages clinically and radiologically, it is still an efficient alternative to anterior cervical plating, especially in single-level fusion, due to the extra cost and plate-related complications.

In the present study, we did not observe any significant difference in functional outcomes (JOA and NDI scores), fusion rate, and ASD between group ZP and PC. The safety and effectiveness of the Zero-P spacer for 3-level ACDF surgery were validated. In the authors’ opinion, with respect to the sagittal alignment, long-segment fusion using a Zero-P spacer was preferable in patients who had a good preoperative cervical lordosis (C2-C7 Cobb > 10°).

There were several limitations in this study. First, this was a single-center study. The number of patients included in this study was relatively small. Second, the follow-up duration was not long. Third, the analyses of spinal sagittal balance, including the thoracic and lumbar spine, were omitted, due to the lack of whole-spine radiographs. Fourth, the fusion rate was only evaluated on X-ray films, not on CT scans; the actual fusion rate might be overestimated. Fifth, ACDF without plating is not analyzed. Although this technique is very rare nowadays, the involvement of this surgical series of patients could add more value to our results. Finally, this study was performed retrospectively, which might lead to some bias. A multicenter, long-term follow-up study might be needed for the further clarification of our findings.

## 5. Conclusions

Zero-P spacers used in 3-level ACDF surgery could provide equivalent clinical outcomes and a lower rate of postoperative dysphagia, compared to a traditional plate and cage system. However, our results showed that it was inferior to a cervical plate in terms of sagittal alignment reconstruction for 3-level fixation. We recommend applying Zero-P spacers for 3-level ACDF in patients with good preoperative cervical lordosis (C2-C7 Cobb angle > 10°), in order to restore and maintain physiological curvature of the cervical spine postoperatively.

## Figures and Tables

**Table 1:** Bazaz grading system for dysphagia.

Symptom Severity	Liquid Food	Solid Food
None	None	None
Mild	None	Rare
Moderate	None or rare	Occasionally (only with specific food)
Severe	None or rare	Frequent (majority of solids)

**Table 2:** Demographics of subjects.

Variables	Group ZP (Zero-P)	Group PC (Plate & Cage)	*P*
No.	23	21	-
Sex (M/F)	13/10	11/10	0.783
Age (y)	50.3 ± 6.8	49.5 ± 7.7	0.546
Operation time (min)	112.5 ± 15.1	117.3 ± 18.6	0.117
Blood loss (mL)	82.4 ± 11.7	88.9 ± 14.1	0.169
FU time (mo)	30.1 ± 4.4	31.4 ± 5.1	0.223
Fused segments			
C3-C6	10	11	0.555
C4-C7	13	10	

FU indicates follow-up.

**Table 3:** Clinical outcomes of subjects.

Variables	Group ZP	Group PC	*P*
	(Zero-P)	(Plate & Cage)	
JOA score			
Preop	8.8 ± 0.8	9.1 ± 0.9	0.743
3-mo Postop	13.3 ± 1.1	13.1 ± 0.9	0.811
Last FU	14.1 ± 1.3	13.9 ± 1.0	0.636
NDI score			
Preop	13.5 ± 2.6	13.8 ± 2.1	0.677
3-mo Postop	5.5 ± 1.4	5.8 ± 1.1	0.725
Last FU	7.1 ± 2.1	7.7 ± 1.8	0.554
Dysphagia			
48-h	8/23 (34.8%)	8/21 (38.1%)	0.82
3-mo	1/23 (4.3%)	6/21 (28.6%)	0.028
6-mo	0/23 (0%)	4/21 (19.0%)	0.028

FU indicates follow-up; JOA, Japanese Orthopaedic Association; NDI, Neck Disability Index.

**Table 4:** Radiological outcomes of subjects.

Variables	Group ZP	Group PC	*P*
	(Zero-P)	(Plate & Cage)	
Fusion rate (24 mo)	21/23(91.3%)	20/21(95.2%)	0.605
ASD (24 mo)	3/23(13.0%)	3/21(14.3%)	0.905
C2-C7 Cobb (°)			
Preop	10.8 ± 8.1	11.1 ± 7.9	0.331
Postop	15.7 ± 7.4	24.8 ± 8.1	0.011
Last FU	12.5 ± 5.7	18.6 ± 6.9	0.023
Fused segment Cobb (°)			
Preop	6.1 ± 6.8	5.9 ± 7.9	0.667
Postop	12.4 ± 7.1	19.8 ± 8.2	0.014
Last FU	9.5 ± 6.6	13.9 ± 7.4	0.025
Fused segment disc wedge (°)			
Preop	5.8 ± 5.1	6.1 ± 6.9	0.564
Postop	11.3 ± 7.2	18.1 ± 7.9	0.012
Last FU	8.1 ± 6.8	13.6 ± 7.5	0.021
C2-C7 SVA (mm)			
Preop	28.4 ± 11.3	30.1 ± 14.4	0.248
Postop	30.2 ± 14.6	28.2 ± 15.7	0.145
Last FU	31.7 ± 13.9	29.6 ± 16.5	0.233

FU indicates follow-up; ASD, adjacent segment degeneration; SVA, sagittal vertical axis.

**Table 5:** Comparison of sagittal alignment by subgroups.

Subgroups	Variables	Group ZP	Group PC	*P*
		(Zero-P)	(Plate & Cage)	
Preop C2-C7 Cobb ≤ 10°	C2-C7 Cobb (°)			
	Preop	4.9 ± 4.1	5.2 ± 4.7	0.516
	Postop	9.7 ± 5.8	17.8 ± 6.5	0.009
	Last FU	7.6 ± 6.6	15.2 ± 8.9	0.011
	Fused segment Cobb (°)			
	Preop	3.1 ± 3.8	2.9 ± 3.9	0.712
	Postop	8.3 ± 6.5	14.8 ± 7.6	0.01
	Last FU	6.4 ± 5.7	13.1 ± 7.0	0.013
	Fused segment disc wedge (°)			
	Preop	2.8 ± 3.1	3.0 ± 2.9	0.411
	Postop	7.5 ± 4.2	12.7 ± 4.9	0.015
	Last FU	5.5 ± 3.9	11.0 ± 5.5	0.008
Preop C2-C7 Cobb > 10°	C2-C7 Cobb (°)			
	Preop	15.8 ± 6.2	16.0 ± 5.9	0.451
	Postop	24.7 ± 11.7	28.8 ± 10.3	0.092
	Last FU	21.1 ± 10.3	23.5 ± 11.6	0.121
	Fused segment Cobb (°)			
	Preop	10.6 ± 4.1	11.9 ± 5.0	0.685
	Postop	18.8 ± 5.1	22.3 ± 6.2	0.146
	Last FU	15.9 ± 6.0	17.3 ± 8.8	0.083
	Fused segment disc wedge (°)			
	Preop	8.8 ± 5.3	9.1 ± 6.3	0.541
	Postop	17.3 ± 6.1	20.1 ± 5.8	0.089
	Last FU	14.1 ± 5.8	15.3 ± 6.6	0.107

FU indicates follow-up.

## Data Availability

The data that support the findings of this study are available from the corresponding author upon reasonable request.
